# Using machine learning to determine the time of exposure to infection by a respiratory pathogen

**DOI:** 10.1038/s41598-023-30306-7

**Published:** 2023-04-01

**Authors:** Kartikay Sharma, Manuchehr Aminian, Tomojit Ghosh, Xiaoyu Liu, Michael Kirby

**Affiliations:** 1 Department of Computer Science, Colorado State University, Fort Collins, CO USA; 2 Department of Mathematics, Colorado State University, Fort Collins, CO USA; 3 Department of Computer Science, University of Maryland, College Park, MD USA; 4 Department of Mathematics, California State Polytechnic University, Pomona, CA USA

**Keywords:** Diagnostic markers, Predictive markers, Prognostic markers, Computational biology and bioinformatics, Applied mathematics, Scientific data

## Abstract

Given an infected host, estimating the time that has elapsed since initial exposure to the pathogen is an important problem in public health. In this paper we use longitudinal gene expression data from human challenge studies of viral respiratory illnesses for building predictive models to estimate the time elapsed since onset of respiratory infection. We apply sparsity driven machine learning to this time-stamped gene expression data to model the time of exposure by a pathogen and subsequent infection accompanied by the onset of the host immune response. These predictive models exploit the fact that the host gene expression profile evolves in time and its characteristic temporal signature can be effectively modeled using a small number of features. Predicting the time of exposure to infection to be in first 48 h after exposure produces BSR in the range of 80–90% on sequestered test data. A variety of machine learning experiments provide evidence that models developed on one virus can be used to predict exposure time for other viruses, e.g., H1N1, H3N2, and HRV. The interferon $\alpha /\beta$ signaling pathway appears to play a central role in keeping time from onset of infection. Successful prediction of the time of exposure to a pathogen has potential ramifications for patient treatment and contact tracing.

## Introduction

The main objective of this paper is to determine when an infected host was exposed to a respiratory pathogen. To accomplish this, we employ machine learning to construct a predictive model that estimates the amount of time that has elapsed since the host has been exposed. This data driven approach uses a clinical challenge data set comprised of time-stamped gene expression data from 7 experiments across 4 respiratory viruses [two subtypes of Influenza A (H1N1, H3N2), Respiratory Syncytial Virus (RSV), and Human Rhinovirus (HRV)]^1,2^. Individual samples have time labels measured from the exact time of exposure to infection, i.e., the recorded time of inoculation with the challenge virus. In addition, each host has subsequent measurements approximately 8 h apart with exact intervals being study dependent. The objective of the resulting model is to estimate how long it has been since a host was first exposed, i.e., the time of the onset of the immune response, which we refer to as the “time of exposure” (ToE) problem. Note that in this study we only consider subjects who tested positive for virus.

Our modeling process begins by determining small sets of time-dependent biomarkers that can be used to discriminate between different time points over the course of infection. These biomarker sets discriminate across the 36 pairs of nine possible time values (“bins”) at 8 and 24 h of resolution. The resulting gene sets are then used to build a family of classifiers that can estimate the ToE. Our results suggest that there is sufficient signal in the seven data sets to roughly approximate the time of infection using machine learning algorithms. The relevant question of whether this type of analysis may be practical for the treatment of patients requires additional work by clinicians and is outside the scope of this investigation.

Common practice for model building requires that the number of datapoints used to build the model should be much larger than the number of learnable parameters in the model, otherwise one runs the risk of overfitting. This occurs when a model is built which successfully describes the training data, but fails to make accurate predictions on previously unseen data; essentially “memorizing” the training set and failing to have predictive power when new data is seen. In the case of image or signal processing, this is not a major issue, since extremely large databases of images and audio exist.

However, when dealing with “omics” data, the analogue of an image is the measurements of all potential biomarkers of interest in an individual at a given time pre- or post-infection, and the corresponding “image resolution” is the number of biomarkers being measured. In the case of RNA transcriptomics, the number of biomarkers is on the order of tens of thousands, while the most focused classification tasks may have tens or hundreds of datapoints at most. This poses a fundamental challenge for omics analyses, since experimental data is typically expensive to obtain. Hence, much of our work is focused on applying techniques from sparse optimization to first learn small collections of discriminatory biomarkers aimed towards analyzing the time of exposure to infection, then study the performance of classifiers on test data, where these classifiers now focus only on these collections of biomarkers.

Sparsity-promoting machine learning techniques have the potential to identify biomarkers that provide insight into the biological processes involved in the host immune response to infection^3–5^. In particular, one may endeavor to identify all of the biomarkers that are discriminatory for a given host immune response using *Iterative Feature Removal*, a repeated application of the sparse removal of top biomarkers until none remain^6^. This approach lends itself well to construction of predictive models, but also provides a potentially comprehensive picture that might lead to biological insights, e.g., in Lyme disease^7^, and influenza^8^; or more generally, to data driven biological pathway analysis^9^. Of specific interest to us in this investigation is the fact that the discriminatory biomarkers associated with respiratory infections appear to be highly time-dependent^8^. Further, they are potentially capable of predicting severity of symptoms, including host virus shedding, at the earliest stages of infection. As we shall show, this infection clock that is inherent in the immune response is effectively captured by the biological signal in the gene expression data, and provides a mechanism to determine the time of exposure to infection.

## Results

**Figure 1:**
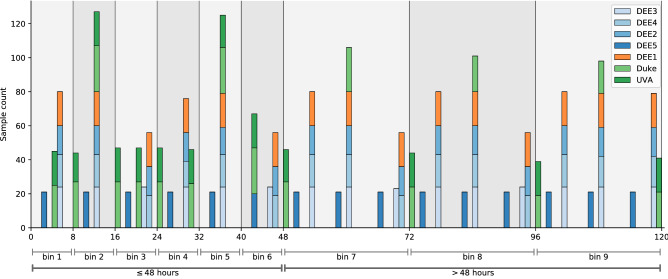
Time distribution of samples in the data set GSE73072 within the first 5 days after inoculation, grouped by study. Categorical labels are assigned as illustrated; either for the nine-class problem (“bin 1” through “bin 9”) or the two-class problem (“$\le 48$ h’ or “$>48$ h”). For example, bin 1 contains data in the time interval (0, 8] h, bin 2 from the interval (8, 16], and so on.

**Figure 2:**
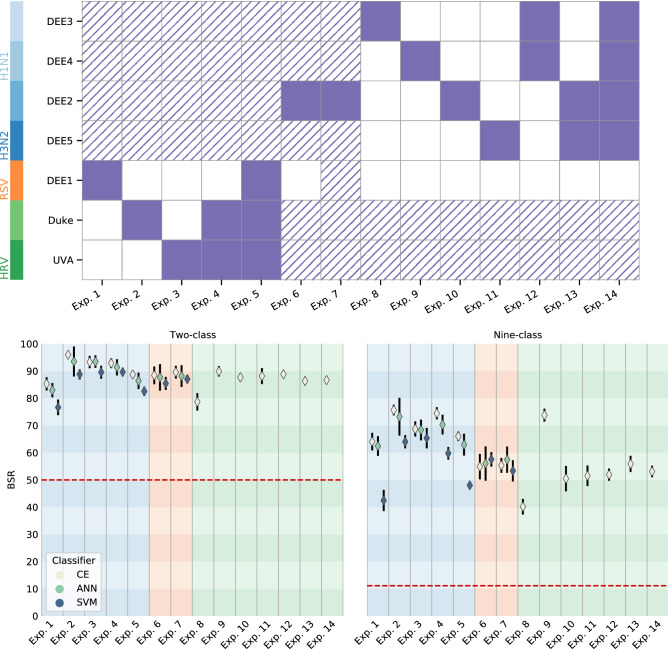
**Top:** Illustration of data involved in each experiment during the feature discovery (striped) and feature validation (solid) phases. Experiments 1–5 learn features on influenza data sets. Experiments 6–7 investigate effects of including RSV data during feature discovery. Experiments 8–14 learn features on HRV data sets. **Bottom:** Summarized classification results for all experiments. Columns are shaded based on experiment theme—Influenza features (blue), effect of RSV data (orange), HRV features (green). Results are shown for both the nine-class problem (predict the correct time window), and two-class problem (predict time of infection as less than or greater than 48 h) in terms of BSR. Neural net classifiers [Centroid-Encoder (CE), Artificial Neural Networks (ANN)] generally outperform a multiclass linear classifier Support Vector Machines (SVM), but all models have predictive power with the learned features. A baseline BSR for random guessing (red dashed line) is shown in each case.

The data used in this investigation consist of a collection of seven human challenge studies available via the NCBI GEO Accession number GSE73072. In total there are 148 subjects with approximately 20 time points per subject; (further details are available in^1^). Our first goal is to identify time-dependent features (i.e., gene expression via microarray probes) predictive of the time of infection to a respiratory virus. Because of variations in the sample times in the human challenge studies, the data are binned based on their time labels into one of six 8-h bins in the first 48 h post-inoculation, or a 24-h bin between hours 48 through 120 (see Fig. 1).

We approach feature discovery on a per-virus basis, with the guiding question that the host-pathogen dynamics may vary enough between each pathogen to elicit distinct predictive biomarkers. Details of the Experiments broken down by training and test sets are shown in Fig. 2. The experiments are arranged in columns, while the studies are arranged in rows, ordered by the challenge virus. Each experiment follows a two step process. First, we select features using our machine learning techniques on the training data. Second, we evaluate these selected features on the test data. Data are included as part of feature selection if marked with stripes and included in feature evaluation if marked solid. While feature/biomarker mapping is not an issue because of a common microarray platform used throughout, batch effects by study can be seen and are corrected on a per-subject basis.

Experiments 1–5 select features using the four influenza-based studies labeled DEE2-DEE5. Feature testing is done using most combinations of the remaining three data sets, which are studies with subjects challenged by HRV or RSV. Performance can be seen in the bottom panels of Fig. 2. Two-class performance (Fig. 2, bottom left) sees performance on the test data to be between 75 and 95% Balanced Success Rate (BSR). Approaches based on neural networks generally outperform a linear model (Linear SVM) by approximately ten percentage points. On the more challenging nine-class problem (Fig. 2, bottom right), to predict the specific label (time bin), performance of selected features are 40–75%. Neural network approaches are seen to further outperform a multiclass linear model, but all classifiers still greatly outperform a baseline of true random guessing (the baseline of 11.1% for a nine-class problem is illustrated).

**Figure 3:**
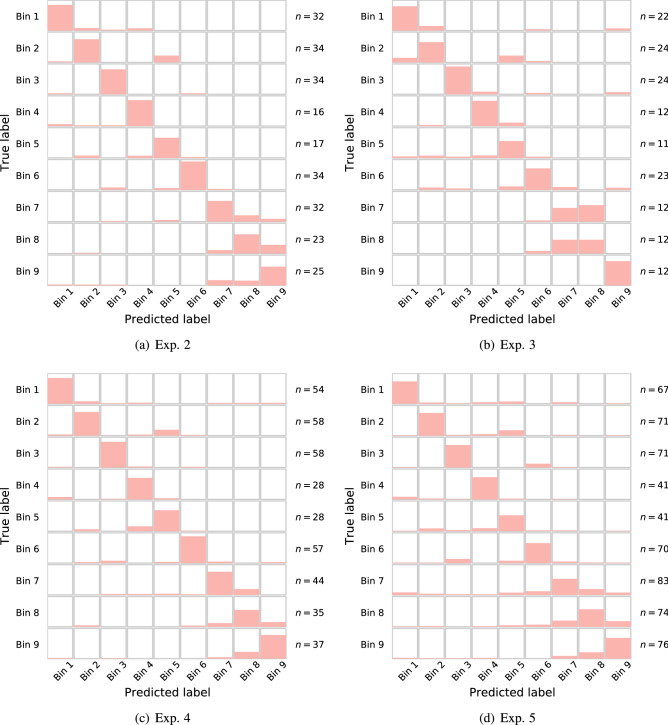
Visualization of nine-class confusion matrices for experiments 2–5, studying performance of influenza features in the time of infection prediction. The height of the bars for each bin corresponds to the number of samples classified in that bin, normalized to the total number of samples in that bin for that experiment (number of samples in each bin shown on right). A diagonally dominant pattern illustrates most test samples were correctly classified, and is synonymous with higher BSR values. Note that misclassifications (off-diagonals) typically are highest in adjacent time bins; demonstrating continuity in the process.

Experiments 8–14 are similar to Experiments 1–5, but features are selected using HRV data (denoted “Duke” and “UVA”), and evaluated using the Influenza studies DEE2-DEE5. In view of the results from Experiments 1–7 (Experiments 6 and 7 discussed below) we focused on CE for these experiments. Performance for the two-class problem is very consistent, with a median BSR of 88%, with the outlier on Experiment 8 (which treats DEE3, an H1N1 study, as the test set). Performance for the nine-class problem has a median BSR of 52% across these experiments, with the poor outlier performance on Experiment 8 (40%), and positive outlier for Experiment 9 (74%, test on DEE4). Overall, performance of features was consistent for both influenza or HRV-based features, with reduced accuracy for the nine-class problem in applying HRV-based features to other virus test data. See Fig. 3 for a visualization of binwise confusion matrices from the classification experiments associated with experiments 2–5.

Finally, Experiments 6 and 7 investigate how the feature selection process may depend on exclusion or inclusion of the same viral pathogen during the feature selection. Experiment 6 includes all non-RSV studies as part of the feature selection, and studies performance with the DEE2 study. Experiment 7 repeats this, but includes DEE1, the sole RSV study, as part of the feature selection process, with an understanding that the pathogen dynamics with RSV may be somewhat distinct from influenza or HRV. One might expect to see performance decrease as a result of the inclusion of this data, but no conclusive trend can be seen from the results. All classifiers see a 1 or 2 percentage point increase with the inclusion of DEE1 in the feature selection, but the performance for the nine-class problem is mixed.

### Analysis of features

We studied the biological relevance of features by comparing against the GSEA (Gene Set Enrichment Analysis) MSigDB (Molecular Signatures database)^10,11^, which includes canonical pathways from KEGG, REACTOME, and BIOCARTA databases (referred to as “C2: curated gene sets”), as well as gene sets identified on other gene expression data sets (“C7: immunologic signature gene sets”). While sophisticated pathway-based analyses are available via proprietary software which can give deeper insight in to underlying biological processes, our purpose here is to simply highlight pathways identified as important by our data driven analysis to lend credence to the machine learning algorithms.

Table 1 summarizes recurring gene sets from MSigDB which highly overlap with our feature selection process. While the feature selection process itself works at the level of individual biomarkers (microarray probes), the table shows the highest scoring pathways (see “Methods” section). Gene sets which are scored highly in more time bins in this table have significant overlap with feature-sets that best describe the time evolution of the immune system. This is done by time bin for the nine-class problem, so a maximum of 9 is possible for the number of time bins. Further details are available in the “Methods” section, and Supplementary Material.

We observe several interferon-associated pathways showing a major presence as the result of the feature extraction process. Interferon $\alpha$/$\beta$ signaling and interferon $\gamma$ signaling pathways, as well as the IL6 pathway, appear to play a major role throughout. Several gene sets associated with the MSigDB “C7: immunologic signature gene sets” are present, corresponding to studies GSE6269—a study of pediatric patients with Influenza A^12^, and GSE34205—a similar study of children hospitalized with acute RSV or influenza infections^13^. This suggests an interesting connection to be explored in future work.

**Table 1:** Top GSEA gene sets associated with our feature sets.

Gene set name	# bins
REACTOME_INTERFERON_ALPHA_BETA_SIGNALING	9
BIOCARTA_AHSP_PATHWAY	7
REACTOME_INTERFERON_GAMMA_SIGNALING	6
GSE6269_HEALTHY_VS_FLU_INF_PBMC_DN	6
GSE6269_FLU_VS_STREP_PNEUMO_INF_PBMC_UP	6
REACTOME_IL_6_SIGNALING	5
GSE34205_HEALTHY_VS_FLU_INF_INFANT_PBMC_DN	4
REACTOME_METABOLISM_OF_PORPHYRINS	4
REACTOME_NFKB_ACTIVATION_THROUGH_FADD_RIP1_PATH.	3
BIOCARTA_NEUROTRANSMITTERS_PATHWAY	3
REACTOME_REGULATION_OF_IFNG_SIGNALING	2
BIOCARTA_DNAFRAGMENT_PATHWAY	2
REACTOME_EXTRINSIC_PATHWAY_FOR_APOPTOSIS	2
BIOCARTA_TCAPOPTOSIS_PATHWAY	2
KEGG_RENIN_ANGIOTENSIN_SYSTEM	2

The number of time bins, designated “$\#$ bins” represents the frequency that the gene set appeared in the “top ten” gene sets for a given time bin. Gene sets present in fewer than 2 time bins are omitted.

**Table 2:** Number of features selected for each pair-wise time bin experiment using the six studies.

Bins	No. features	Bins	No. features	Bins	No. features
Bin1 vs Bin2	65	Bin2 vs Bin7	60	Bin4 vs Bin8	170
Bin1 vs Bin3	190	Bin2 vs Bin8	90	Bin4 vs Bin9	135
Bin1 vs Bin4	200	Bin2 vs Bin9	85	Bin5 vs Bin6	65
Bin1 vs Bin5	40	Bin3 vs Bin4	160	Bin5 vs Bin7	120
Bin1 vs Bin6	170	Bin3 vs Bin5	160	Bin5 vs Bin8	200
Bin1 vs Bin7	30	Bin3 vs Bin6	165	Bin5 vs Bin9	85
Bin1 vs Bin8	75	Bin3 vs Bin7	110	Bin6 vs Bin7	95
Bin1 vs Bin9	55	Bin3 vs Bin8	30	Bin6 vs Bin8	155
Bin2 vs Bin3	130	Bin3 vs Bin9	140	Bin6 vs Bin9	115
Bin2 vs Bin4	120	Bin4 vs Bin5	90	Bin7 vs Bin8	105
Bin2 vs Bin5	150	Bin4 vs Bin6	150	Bin7 vs Bin9	105
Bin2 vs Bin6	195	Bin4 vs Bin7	175	Bin8 vs Bin9	150

## Discussion

Controlling respiratory infections and their spread is clearly a significant global health challenge. The death toll resulting from all respiratory including influenza, HRV and pneumonia has been estimated to be approximately 5 million people per year with such illnesses being identified as the leading cause of death in children^14^. Models have determined that, on average, some 450,000 deaths can be attributed to influenza each year^15^. These numbers reflect the average seasonal threat. The worst case scenarios associated with global pandemics are extreme. It has been reported that the 1918 Spanish flu pandemic had over 40 million deaths, or approximately 1 in 50 people worldwide^16^. If a modern pandemic had the same mortality rate the end result would be a staggering 150 million deaths.

Antiviral drugs have emerged as a powerful tool for the early treatment of viral infections, and are particularly effective for respiratory infections such as influenza^17,18^. Clinical evidence suggests that the administration of antivirals is most effective during the first 48 h post infection with influenza^19–21^. While rapid tests administered in the clinic to test for influenza have high positive predictive value^22^ they do not determine the actual time-of-infection to the pathogen. Hence, the effectiveness of the administration of antivirals could be enhanced if the ToE could be accurately estimated in the clinic. The results of this paper suggest the possibility of a predictive model that can be administered by a clinician to determine the amount of time that has elapsed since exposure to the respiratory virus. This could result in considerable savings, and in particular help mitigate seasonal shortages in the supply of antivirals.

Our preliminary results here are encouraging with BSR values at 80–90% on sequestered test samples. Currently, our best results, when predicting to within 8 h, range from 50 to 80% using CE, with the highest performance when validating features extracted on influenza data (experiments 1–5; see 2). The results here affirm it is possible to chart out the progress of immune markers and map them back to the time elapsed since exposure with good success. It is an open research question if these classification rates are limited by actual temporal resolution of biological processes, or inherent noise limitations in biological data. These issues may be resolved in the future as additional data sets become available.

A dividend of this investigation is the identification of a collection of biomarkers that capture the time evolving response of the immune system to infectious disease. Further analysis using systems biology approaches is essential to elucidate biological processes associated with ToE and the role of the identified biomarkers and pathways. This could potentially result in the development of targeted panels that could be deployed for pandemic prevention.

In this study we have not explored the subjects who were asymptomatic after inoculation. It will be interesting to do this, and attempt to determine how the host immune response for these individuals provides insight into their resistance to infection.

## Methodology

In this section we describe the clinical data set, data preprocessing, feature selection process, classifiers used, and design for the machine learning experiments.

### Experimental setup

The data we study is a collection of seven clinical studies available via the NCBI Gene Expression Omnibus, Series GSE73072. The details of these studies can be found in^1^, but we briefly summarize here and in Fig. 1.

Each of the seven studies enrolled individuals to be infected with one of four viruses associated with a common respiratory infection. Studies DEE2-DEE5 challenged participants with H1N1 or H3N2. Studies “Duke” and “UVA” challenged participants with HRV, while ”DEE1” challenged individuals with RSV.

In all cases, individuals had blood samples taken at regular intervals every 4–12 h both prior to and after infection; see Fig. 1 for details. Specific time points are measured as hours since infection and vary by study. In total, 148 human subjects were involved with approximately 20 sampled time points per person. Blood samples were run through undirected microarray assays. CEL data files available via GEO were read and processed using RMA (Robust Multi-array Average) normalization through use of several Bioconductor packages^23^ producing expression values across 22,277 microarray probes.

To address the time of infection question, we separate the training and test samples into 9 bins in time post-inoculation, each with a categorical label; see Fig. 1. The first six categories correspond to disjoint 8-h intervals in the first 2 days after inoculation, and the last three categories are disjoint 24-h intervals from hours 48 to 120 h. In addition to this 9-class classification problem, we also studied a “relaxed” binary prediction problem of whether a subject belongs to the early phase of infection (time of inoculation $\le$ 48 h) or later phase (time of infection > 48 h). Results for this binary classification are inferred from the 9-class problem, i.e., if a classified label is associated to a time in the first 2 days, it is considered correctly labeled.

After the data is processed, we apply the following general pipeline for each of the 14 experiments enumerated in Fig. 2 (top panel):

1. Partition the data into training and testing sets based on the classification experiment.
2. Normalize the data to correct for batch effects seen between subjects (e.g., using the linear batch normalization routine in the *limma* package^24^).
3. Identify comprehensive sets of predictive features using the Iterative Feature Removal (IFR) approach^6^, which aims to extract all discriminatory features in high-dimensional data with repeated application of sparse linear classifiers such as Sparse Support Vector Machines (SSVM).
4. Identify network architectures and other hyperparameters for Artificial Neural Networks (ANN) and Centroid Encoder (CE) by utilizing a five-fold cross-validation experiment on the training data.
5. Evaluate the features identified from the step 3 on the test data. This is done by training and evaluating a new model using the selected features with a leave-one-subject-out cross validation scheme on the test study. The metric used for evaluation is BSR; throughout this study we utilize BSR as a balanced representation of performance accounting for imbalanced class sizes while being easy to interpret.

### Feature selection

For each of the training sets illustrated in Fig. 2 (top panel; stripes), features are selected using the IFR algorithm, with an SSVM classifier. This is done separately for all pairwise combinations of categorical labels (time bins); a 9-class experiment leads to 9-choose-2 = 36 pairwise combinations. So, for each of these 36 combinations of time bins, features are selected using the following steps.

First, the input data to IFR algorithm is partitioned into training and validation set. Next, sets of features that produce high accuracy on the validation set are selected iteratively. In each iteration, features that have previously been selected are masked out, so that they’re not used again. The feature selection is halted once the predictive rates on the validation data drops below a specified threshold. This results in one feature-set for a particular training-validation set of the input data.

Next, more training-validation partitions are repeatedly sampled, and the feature selection, as described above, is repeated for each partition-set; this results in a different feature-set for each new partition-set. Then, these different feature-sets are combined by applying a set union operation and the frequency of each individual feature is tracked if they are discovered in multiple feature-sets. The feature frequency is used to rank the features; the more a particular feature is discovered, the more important it is.

The size of this combined feature-set, although about 5–20% of the original feature-set size, is still often large for classification, so a last step we reduce the size of this feature-set. This is done by performing a grid-search using a linear SVM (without sparsity penalty) on the training data, taking the top *n* features, ranked by frequency, which maximize the average of true positive rates on every class, or BSR. Once the features have been selected, we perform a more detailed leave-one-subject-out classification for the experiments described in the Results and visualized in Fig. 2 using the classifiers described in “Methods” section.

### Scoring of gene sets

Feature selection produced 36 distinct feature-sets, coming from all distinct choices of two time bins from the nine labels possible. To address the question of commonality or importance of features selected on a time bin for a specific pathway, we implemented a heuristic scoring system. For a fixed time bin (say, bin1) and a fixed feature-set (say, bin1_vs_bin2; quantities summarized in Table 2) the associated collection of features was referenced against the GSEA MSigDB. This database includes both canonical pathways and gene sets as the result of data mining—we refer to anything in this collection generically as a “gene set.” A score for each MSigDB gene set was assigned for a given feature-set (bin1_vs_bin2) based on the ratio of features in the feature-set which appear in the gene set. For instance, a score of 0.5 for hypothetical GENE_SET_A for feature-set bin1_vs_bin2 represents the fact that 50% of the features in GENE_SET_A are present in bin1_vs_bin2.

A score for pathway on a time bin by itself was defined as the sum of the scores for that pathway on all feature-sets related to it. Continuing the example, a score for GENE_SET_A on bin1 would be the sum of the scores for GENE_SET_A for feature-set bin1_vs_bin2, bin1_vs_bin3, all the way up to bin1_vs_bin9, with equal weighting.

Certainly, there are several subtle statistical and combinatorial questions relating to this procedure. Direct comparison of pathways and gene sets is challenging due their overlapping nature (features may belong to multiple gene sets). The number of features associated with a gene set can vary anywhere from under 10, to over 1000 and may complicate a scoring system based on percentage overlap, such as ours. Attempting to use a mathematically or statistically rigorous procedure to account for these, and other potential factors is a worthy exercise, but we believe our heuristic is sufficient for an explainable high-level summary of the composition of the feature-sets found.

### Classification

In this section we describe the classifiers and how they are applied for the classification task. We also describe how the feature-sets are adapted to different classifiers.

After feature selection, we evaluate the features on test sets based on successful classification in the nine time bins. For each experiment shown in Fig. 1, we use the feature-sets extracted on its training set and evaluate the models using leave-one-subject-out cross validation on the test set. Each experiment is repeated 25 times to capture variability. For the binary classifiers—SSVM and linear SVM—we used a multiclass method, with each of its ${9 \atopwithdelims ()2}$ pairwise models using respective feature-sets. On the other hand, we used a single classification model for ANN and CE because these models can handle multiple classes. The feature-set for these models are created by taking a union of ${9 \atopwithdelims ()2} = 36$ pairwise feature-sets.

#### Metrics

*Balanced Success Rate (BSR)* Throughout the Results section, we report predictive power in terms of BSR. This is a simple average of true positive rates for each of the categories. The BSR serves as a simple, interpretable metric especially when working with imbalanced data sets and gives a holistic view of classification performance that easily generalizes to multiclass problems. For example, if true positive rates in a 3-class problem were $TPR_1 = 95\%$, $TPR_2 = 50\%$, and $TPR_3 = 65\%$, the BSR for the multiclass problem would be $(TPR_1 + TPR_2 + TPR_3)/3 = 70\%$.

#### Multiclass classification using binary classifiers

We implement a pairwise model (or “one-vs-one” model) for training and classification to extend the binary classifiers described below (ANN and CE do not require these). For a data set with *c* unique classes, *c*-choose-2 models are built using the relevant subsets of the data. Learned model parameters and features selected for each model are stored and later used when discriminatory features are needed in the test phase.

After training, classification is done by a simple voting scheme: a new sample is classified by all *c*-choose-2 classifiers and assigned the label that had the plurality of the vote. If a tie occurs, the class is decided by an unbiased coin flip between the winning labels. In a nine-class problem, this corresponds to 36 classifiers and feature-sets being selected.

*Linear SVM* For a plain linear SVM model, the implementation in the scikit-learn package in Python was used^25^. While scikit-learn also has built-in support to extend this binary classifier to multiclass problems, either by one-vs-one or one-vs-all approaches, we only use it for binary classification problems, or for binary sub-problems of a one-vs-one scheme for a multiclass problem. The optimization problem was introduced by^26^ and requires the solution to

1 $$\begin{aligned} \begin{aligned} \textrm{min}_{w,b} \;&||w||_2^2 \quad \text {subject to} \\&y^i ( w \cdot x^i - b ) \ge 1, \quad \text { for all } i \end{aligned} \end{aligned}$$

where $y^i$ represent class labels assigned to $\pm 1$, $x^i$ represent vector samples, *w* represents the weight vector and *b* represents a bias (a scalar shift). This approach has seen widespread use and success in biological feature extraction^27,28^.

*Sparse SVM (SSVM)* The SSVM problem replaces the 2-norm in the objective of equation 1 with a 1-norm, which is understood to promote sparsity (many zero coefficients) in the coefficient vector ${\textbf{w}}$. This allows one to ignore those features and is our primary tool for feature selection when coupled with Iterated Feature Removal^6^. Arbitrary *p*-norm SVM were introduced in^29^ and $\ell _1$-norm sparse SVM were further developed for feature selection in^6,30,31^.

#### Multiclass classifiers

After a standard one-hot encoding scheme, inherently multiclass methods (here: neural networks) do not need to be adapted to handle a multiclass problem as with linear methods, nor is there a straightforward way to encode the use of time-dependent features in passing new data forward through the neural network; this would be begging the (time of infection) question. Instead, for these methods, we simply take the union of all “pairwise” features built to classify pairs of time bins, then allow the multiclass algorithm to “learn” any necessary relationships internally. The specifics of the neural networks are described below.

*Artificial Neural Networks (ANN)* We apply a standard feed-forward neural network trained to learn the labels of the training data. In all the classification tasks, we used two hidden layers with 500 ReLU activation in each layer. We used the whole training set to calculate the gradient of the loss function (Cross-entropy) while updating the network parameters using Scaled Conjugate Gradient Descent (SCG); see^32^.

*Centroid-Encoder (CE)* This is a variation of an autoencoder which can be used for both visualization and classification purposes. Consider a data set with *N* samples and *M* classes. The classes denoted $C_j, j = 1, \dots , M$ where the indices of the data associated with class $C_j$ are denoted $I_j$. We define centroid of each class as $c_j=\frac{1}{|C_j|}\sum _{i \in I_j} x_i$ where $|C_j|$ is the cardinality of class $C_j$. Unlike autoencoder, which maps each point $x_i$ to itself, CE will map each point $x_i$ to its class centroid $c_j$ by minimizing the following cost function over the parameter set $\theta$:

2 $$\begin{aligned} \begin{aligned} {\mathscr {L}}_{ce}(\theta )=\frac{1}{2N}\sum ^M_{j=1} \sum _{i \in I_j}\Vert c_j-f(x_i; \theta ))\Vert ^2_2 \end{aligned} \end{aligned}$$

The mapping *f* is composed of a dimension reducing mapping *g* (encoder) followed by a dimension increasing reconstruction mapping *h* (decoder). The output of the encoder is used as a supervised visualization tool^33^, and attaching another layer to map to the one-hot encoded labels and further training by fine-tuning provides a classifier. For further details, see^34^. In all of the classification tasks, we used three hidden layers ($500 \rightarrow 100 \rightarrow 500$) with ReLU activation for centroid mapping. After that we attached a classification layer with one-hot-encoding to the encoder ($500 \rightarrow 100$) to learn the class label of the samples. The model parameters were updated using SCG.

## Supplementary Information

Supplementary Information.

## Data Availability

The data used in this investigation consist of a collection of seven human challenge studies available via the NCBI GEO Accession number GSE73072.
