# Supplementary material for: Using machine learning to determine the time of exposure to infection by a respiratory pathogen
Source: Sci Rep. 2023 Apr 1;13:5340. doi: 10.1038/s41598-023-30306-7 (PMC10067823; doi:10.1038/s41598-023-30306-7)
Supplement: Supplementary file 1 — Supplementary Information. [file 41598_2023_30306_MOESM1_ESM.pdf]

1 Supplemental Material

The tables below detail the “top ten" gene sets per time bin based on the scoring system described in the Methods Section of the main manuscript.

Table 1. bin1

gene_set_name	score	gene_set_size
REACTOME_IL_6_SIGNALING	0.8182	11
REACTOME_REGULATION_OF_IFNG_SIGNALING	0.7143	14
REACTOME_NFKB_ACTIVATION_THROUGH_FADD_RI	0.5833	12
REACTOME_INTERFERON_ALPHA_BETA_SIGNALING	0.5781	64
REACTOME_TRAF3_DEPENDENT_IRF_ACTIVATION_	0.5714	14
ST_TYPE_I_INTERFERON_PATHWAY	0.5556	9
REACTOME_GROWTH_HORMONE_RECEPTOR_SIGNALI	0.5417	24
BIOCARTA_AHSP_PATHWAY	0.5385	13
BIOCARTA_D4GDI_PATHWAY	0.5385	13
REACTOME_INTERFERON_GAMMA_SIGNALING	0.5079	63

Table 2. bin2

gene_set_name	score	gene_set_size
REACTOME_INTERFERON_ALPHA_BETA_SIGNALING	0.7188	64
REACTOME_IL_7_SIGNALING	0.5455	11
REACTOME_IL_6_SIGNALING	0.5455	11
BIOCARTA_CD40_PATHWAY	0.5333	15
REACTOME_INTERFERON_GAMMA_SIGNALING	0.5238	63
GSE6269_HEALTHY_VS_FLU_INF_PBMC_DN	0.5062	160
GSE6269_FLU_VS_STREP_PNEUMO_INF_PBMC_UP	0.4970	169
BIOCARTA_AHSP_PATHWAY	0.4615	13
GSE34205_HEALTHY_VS_FLU_INF_INFANT_PBMC_	0.4500	200
BIOCARTA_TNFR2_PATHWAY	0.4444	18

Table 3. bin3

gene_set_name	score	gene_set_size
REACTOME_SEMA3A_PAK_DEPENDENT_AXON_REPUL	0.4667	15
BIOCARTA_AHSP_PATHWAY	0.4615	13
PID_ERBB_NETWORK_PATHWAY	0.4000	15
REACTOME_TRAF6_MEDIATED_IRF7_ACTIVATION_	0.4000	10
REACTOME_INTERFERON_ALPHA_BETA_SIGNALING	0.3594	64
BIOCARTA_HSP27_PATHWAY	0.3333	15
BIOCARTA_NEUROTRANSMITTERS_PATHWAY	0.3333	6
GSE6269_HEALTHY_VS_FLU_INF_PBMC_DN	0.3187	160
REACTOME_APOPTOSIS_INDUCED_DNA_FRAGMENTA	0.3077	13
BIOCARTA_DNAFRAGMENT_PATHWAY	0.3000	10

Table 4. bin4

gene_set_name	score	gene_set_size
BIOCARTA_AHSP_PATHWAY	0.6154	13
SA_PROGRAMMED_CELL_DEATH	0.5000	12
BIOCARTA_GLYCOLYSIS_PATHWAY	0.4000	10
REACTOME_METABOLISM_OF_PORPHYRINS	0.3571	14
GSE6269_FLU_VS_STREP_PNEUMO_INF_PBMC_UP	0.3491	169
REACTOME_INTERFERON_ALPHA_BETA_SIGNALING	0.3438	64
BIOCARTA_NEUROTRANSMITTERS_PATHWAY	0.3333	6
REACTOME_NFKB_ACTIVATION_THROUGH_FADD_RI	0.3333	12
BIOCARTA_CTLA4_PATHWAY	0.3333	21
REACTOME_INTERFERON_GAMMA_SIGNALING	0.3175	63

Table 5. bin5

gene_set_name	score	gene_set_size
REACTOME_EXTRINSIC_PATHWAY_FOR_APOPTOSIS	0.5385	13
REACTOME_INTERFERON_ALPHA_BETA_SIGNALING	0.4844	64
REACTOME_GABA_SYNTHESIS_RELEASE_REUPTAKE	0.4706	17
BIOCARTA_PML_PATHWAY	0.4706	17
GSE6269_FLU_VS_STREP_PNEUMO_INF_PBMC_UP	0.4556	169
BIOCARTA_TCAPOPTOSIS_PATHWAY	0.4545	11
REACTOME_NFKB_ACTIVATION_THROUGH_FADD_RI	0.4167	12
BIOCARTA_SODD_PATHWAY	0.4000	10
BIOCARTA_LONGEVITY_PATHWAY	0.4000	15
BIOCARTA_DNAFRAGMENT_PATHWAY	0.4000	10

Table 6. bin6

gene_set_name	score	gene_set_size
SA_FAS_SIGNALING	0.4444	9
REACTOME_INTERFERON_ALPHA_BETA_SIGNALING	0.4219	64
REACTOME_CREATION_OF_C4_AND_C2_ACTIVATOR	0.4000	10
REACTOME_IL_6_SIGNALING	0.3636	11
BIOCARTA_TCAPOPTOSIS_PATHWAY	0.3636	11
REACTOME_REGULATION_OF_IFNG_SIGNALING	0.3571	14
GSE6269_HEALTHY_VS_FLU_INF_PBMC_DN	0.3563	160
BIOCARTA_CTL_PATHWAY	0.3333	15
KEGG_CIRCADIAN_RHYTHM_MAMMAL	0.3077	13
REACTOME_EXTRINSIC_PATHWAY_FOR_APOPTOSIS	0.3077	13

Table 7. bin7

gene_set_name	score	gene_set_size
BIOCARTA_AHSP_PATHWAY	1.5385	13
REACTOME_IL_6_SIGNALING	0.9091	11
BIOCARTA_NEUROTRANSMITTERS_PATHWAY	0.6667	6
REACTOME_INTERFERON_ALPHA_BETA_SIGNALING	0.6406	64
GSE6269_HEALTHY_VS_FLU_INF_PBMC_DN	0.6188	160
GSE6269_FLU_VS_STREP_PNEUMO_INF_PBMC_UP	0.5799	169
REACTOME_INTERFERON_GAMMA_SIGNALING	0.4762	63
KEGG_RENIN_ANGIOTENSIN_SYSTEM	0.4706	17
GSE34205_HEALTHY_VS_FLU_INF_INFANT_PBMC_	0.4300	200
REACTOME_METABOLISM_OF_PORPHYRINS	0.4286	14

Table 8. bin8

gene_set_name	score	gene_set_size
REACTOME_METABOLISM_OF_PORPHYRINS	1.1429	14
BIOCARTA_AHSP_PATHWAY	1.0769	13
REACTOME_INTERFERON_ALPHA_BETA_SIGNALING	0.9062	64
GSE6269_HEALTHY_VS_FLU_INF_PBMC_DN	0.7375	160
GSE6269_FLU_VS_STREP_PNEUMO_INF_PBMC_UP	0.6686	169
REACTOME_INTERFERON_GAMMA_SIGNALING	0.6508	63
GSE34205_HEALTHY_VS_FLU_INF_INFANT_PBMC_	0.5900	200
REACTOME_INTERFERON_SIGNALING	0.5723	159
REACTOME_IL_6_SIGNALING	0.5455	11
KEGG_PORPHYRIN_AND_CHLOROPHYLL_METABOLIS	0.5122	41

Table 9. bin9

gene_set_name	score	gene_set_size
BIOCARTA_AHSP_PATHWAY	1.3077	13
REACTOME_INTERFERON_ALPHA_BETA_SIGNALING	0.7031	64
REACTOME_METABOLISM_OF_PORPHYRINS	0.6429	14
GSE6269_HEALTHY_VS_FLU_INF_PBMC_DN	0.5563	160
GSE6269_FLU_VS_STREP_PNEUMO_INF_PBMC_UP	0.5266	169
REACTOME_INTERFERON_GAMMA_SIGNALING	0.4762	63
GSE34205_HEALTHY_VS_FLU_INF_INFANT_PBMC_	0.4650	200
REACTOME_G1_S_SPECIFIC_TRANSCRIPTION	0.4211	19
KEGG_RENIN_ANGIOTENSIN_SYSTEM	0.4118	17
GSE6269_FLU_VS_E_COLI_INF_PBMC_UP	0.4074	162
